# A prospective randomised controlled trial of mechanical axis with soft tissue release balancing vs functional alignment with bony resection balancing in total knee replacement—a study using Stryker Mako robotic arm-assisted technology

**DOI:** 10.1186/s13063-022-06494-4

**Published:** 2022-07-20

**Authors:** Simon W. Young, Nina Zeng, Mei Lin Tay, David Fulker, Christina Esposito, Matthew Carter, Ali Bayan, Bill Farrington, Rupert Van Rooyen, Matthew Walker

**Affiliations:** 1 Department of Orthopaedic Surgery, North Shore Hospital, Auckland, New Zealand; 2 Department of Surgery, University of Auckland, Auckland, New Zealand; 3 Stryker Australia Pty Ltd, St Leonards, NSW Australia; 4 Stryker New Zealand Ltd, Auckland, New Zealand

**Keywords:** Functional alignment, Mechanical alignment, Total knee arthroplasty, Robotic surgery, Randomised controlled trial

## Abstract

**Background:**

Improving the functional outcome following total knee arthroplasty (TKA) by using different alignment techniques remains controversial. The surgical techniques and technologies used so far to obtain these alignments have all suffered from inaccuracies. The use of robotic technology to plan and execute the bony resection provides increased accuracy for these various alignment techniques and may determine which will deliver superior function. Functional alignment (FA) is a newer surgical technique that aims to position the prosthesis with respect to each patients’ specific bony anatomy whilst minimising disruption to the soft tissue envelope. This trial aims to compare the patient and surgical outcomes of FA to the current gold standard surgical technique, mechanical alignment (MA), under randomised and blinded conditions.

**Methods:**

Patients with symptomatic knee osteoarthritis will be prospectively recruited. Following informed consent, 240 patients will be randomised to either a MA surgical technique (the control group) or a FA surgical technique (the intervention group) at a ratio of 4:1 using a random number generator. All patients will undergo computer tomography (CT) based robotic arm-assisted surgery to execute planned implant positioning and alignment with high levels of accuracy. The primary outcome is the forgotten joint score (FJS) at 2 years post-operation. Secondary outcome measures include patient reported outcome measures of post-operative rehabilitation, pain, function and satisfaction, as well as limb alignment, implant revisions and adverse events. Intention-to-treat and per-protocol population analysis will also be conducted. Standardisation of the surgical system and care pathways will minimise variation and assist in both patient and physiotherapist blinding. Ethical approval was obtained from the Northern B Health and Disability Ethics Committee (20/NTB/10).

**Discussion:**

Currently, MA remains the gold standard in knee replacement due to proven outcomes and excellent long-term survivorship. There are many alternative alignment techniques in the literature, all with the goal of improving patient outcomes. This study is unique in that it leverages an advanced analytics tool to assist the surgeon in achieving balance. Both alignment techniques will be executed with high precision using the CT-based robotic arm-assisted surgery system which will minimise surgical variation. This trial design will help determine if FA delivers superior outcomes for patients.

**Trial registration:**

Australia and New Zealand Clinical Trials Registry (ANZCTR), ACTRN12620000009910. Registered on 9 January 2020. ClinicalTrials.gov, NCT04600583. Registered on 29 September 2020.

## Administrative information

**Table:** 

Title {1}	A prospective randomised controlled trial of Mechanical Axis with Soft Tissue Release Balancing vs Functional Alignment with Bony Resection Balancing in Total Knee Replacement – A study using Stryker Mako Robotic-Arm Assisted Technology
Trial registration {2a and 2b}	Australia and New Zealand Clinical Trials Registry (ANZCTR): ACTRN12620000009910 www.clinicaltrials.gov: NCT0460058
Protocol version {3}	Version 3.0, 9th April 2021
Funding {4}	Stryker (Kalamazoo, MI, USA) is funding the data collection and non-standard of care patient management costs
Author details {5a}	A/Prof Simon W Young^1,2^ Dr Nina Zeng^1^ Miss Mei Lin Tay^1,2^ Dr David Fulker^3^ Dr Christina Esposito^3^ Mr Matthew Carter^4^ Mr Ali Bayan^1^ Mr Bill Farrington^1^ Mr Rupert Van Rooyen^1^ Mr Matthew Walker^1^ ^1^Department of Orthopaedic Surgery, North Shore Hospital, Auckland, New Zealand ^2^Department of Surgery, University of Auckland, Auckland, New Zealand ^3^Stryker Australia Pty Ltd, St Leonards, NSW, Australia ^4^Stryker New Zealand Ltd, Auckland, New Zealand
Name and contact information for the trial sponsor {5b}	Stryker New Zealand Ltd (An entity of Stryker (Kalamazoo, MI, USA) Auckland, New Zealand 511 Mount Wellington Highway Mount Wellington 1060 Auckland Tel. + 64 9 573 1890 Email: strykerclinicalresearch@stryker.com
Role of sponsor {5c}	Stryker (Kalamazoo, MI, USA) supports the study design, collection, management and analysis of data and is responsible for HREC requirements. They also provide insurance for the study. There are no terms or conditions to the funding that will impact the interpretation of data or writing the manuscript

## Introduction

### Background and rationale {6a}

Total knee arthroplasty (TKA) is an established treatment for patients with symptomatic end-stage osteoarthritis. The aim of TKA is to provide pain relief and restore function; however, published literature identifies a consistent subset of patients who are not satisfied post-operatively [1–3]. Accuracy of limb alignment, implant position and soft tissue balance are important factors that influence the outcome of TKA [4–7]. It is theorised that modern prosthesis and surgical systems with high precision could assist the surgeon in optimising these factors to an individual patient’s anatomy, which may reduce the dissatisfaction rate following TKA.

Mechanical alignment (MA) has long been the gold standard in TKA and has shown excellent survivorship of 82.3% at 25 years, indicating that most knee replacements will outlast the patient’s lifetime [8]. This technique targets a neutral limb alignment through perpendicular bone resections relative to the mechanical axis of the femur and tibia [9]. It also aims for symmetrical and balanced gaps in flexion and extension, which may require surgical release of the soft tissues [6, 10, 11]. More recently, surgeons have utilised adjustments to bone cuts to achieve balance, such as minor adjustments to femoral rotation for flexion balancing, or leaving up to 3° residual varus in the tibial cut to minimise the need for soft tissue release [12]. Surgeons following these steps often refer to the technique as adjusted mechanical alignment (aMA). A recent publication by Macdessi et al. [13] showed that only 14.6% of arthritic patients have a neutral limb alignment and neutral joint line, as defined by a window of ± 3° and ± 2°, respectively. This indicates that most patients will require surgical adjustments to achieve a goal of MA or aMA, which may alter their native bony and soft tissue anatomy. Furthermore, surgical lengthening of ligaments is a challenging component of the procedure and can be highly variable [14, 15].

For these reasons, surgeons have investigated alternative alignment philosophies to achieve balance in TKA. Kinematic alignment (KA) aims to restore the patient’s native pre-arthritic knee anatomy through symmetrical bone resections relative to the femoral and tibial joint lines [16, 17]. It does not have prescribed alignment boundaries and suggests minimal adjustments to the soft tissue are required if the joint is resurfaced with the implant. However, multiple randomised control trials (RCT) have subsequently found minimal difference in patient outcomes when compared against traditional MA techniques [18]. Arguments for confounders can be made that studies on KA have used a variety of implant designs, surgical technologies with varying degrees of accuracy, and do not always report the corrections in bony morphology or soft tissue corrections [19].

Both MA and KA seek the same outcome: a reliable surgical technique and optimised patient outcome without compromising survivorship. Whilst MA relies on ligamentous adjustments to achieve balance and KA seeks to restore the native bony anatomy through controlled resections, neither considers both aspects together. More recently a technique called functional alignment (FA) was described, which aims to restore the patients native limb alignment and joint line obliquity by adjustments to the implant position based on individual patient bony anatomy and soft tissue balance [20]. The emergence of this technique has coincided with the increasing popularity of image-based robotic arm-assisted surgery, which provides high precision bone resections [21], intraoperative soft tissue laxity assessment allowing for pre-resection balancing [22] and insight into the native anatomy through a pre-operative CT scan [23]. Further, robotic systems that offer haptic control can preserve the soft tissues, particularly the posterior cruciate ligament [23], thereby assisting in the recreation of native kinematics. Whilst long-term data is currently lacking, early cohort studies on robotic arm-assisted TKA following FA principles show promising results [24–26].

There are no published prospective RCTs investigating robotic arm-assisted TKA with FA. Currently, there are two RCTs being conducted in Australia [27, 28] and one at University College London Hospital [29]. All three trials differ in their surgical alignment limits, balancing algorithm and use of assistive technology. The combined results of various trials may help determine the ideal surgical technique for different patient phenotypes [13].

### Objectives {7}

The primary objective of this study is to compare the Forgotten Joint Score (FJS) in MA TKA versus FA TKA at 2 years post-operatively. The FJS is a score that measures the restoration of ‘normal joint feeling’, and the hypothesis is that patients undergoing FA TKA will have a superior score.

The secondary objectives compare the following measures between each cohort:

1. Other patient reported outcomes including: Oxford Knee Score (OKS), International Knee Society Score (IKSS), Knee Injury and Osteoarthritis Outcome Score (KOOS) and Satisfaction
2. Measures of pain throughout the care pathway using the visual analogue scale for pain (VAS Pain), Brief Pain Inventory (BPI) and Pain Sensitivity Questionnaire (PSQ)
3. Post-operative rehabilitation measured through functional tests and range of motion in the operative joint
4. Early recovery data focusing on pain, medication and physiotherapy through recovery data collection form
5. Health-related quality of life assessed through EQ-5D-5L
6. Patient experience assessed through the care pathway using the Net Promoter Score
7. Surgical efficiency by comparing operative times, implant positions and soft tissue laxity measures using the robotic system, in conjunction with pre- and post-operative long leg weight bearing x-rays
8. Complications assessed through adverse events and revision procedures

### Trial design {8}

This study is a prospective, singe-centre, single blinded, randomised controlled trial, where 240 patients will be allocated to robotic arm-assisted TKA following either MA (control group) or FA (intervention group). Participants will be randomly allocated in blocks of four following their eligibility assessment and provision of consent. The study seeks to assess if the interventional surgical technique is superior to the control, where superiority is defined as a patient reported outcome that meets the minimum clinically important difference (MCID) and patient acceptable symptom state (PASS) at 2 years.

## Methods: participants, interventions and outcomes

### Study setting {9}

The study will be conducted in the Orthopaedic Department at North Shore Hospital, Auckland, New Zealand, which falls under the governance of the Waitemata District Health Board. All patients will have surgery, inpatient stays and follow-up at North Shore Hospital or the Elective Surgery Center, which presides within the hospital campus.

### Eligibility criteria {10}

The inclusion criteria are as follows:

- The patient is a male or non-pregnant female between the ages of 40 and 80 years
- The patient requires a primary total knee replacement and is indicated for robotic-assisted surgery
- Patient is deemed appropriate for a cruciate retaining knee replacement
- The patient has a primary diagnosis of osteoarthritis (OA)
- The patient has intact collateral ligaments
- The patient is able to undergo CT scanning of the affected limb
- The patient has signed the study specific, ethics-approved, informed consent ocument
- The patient is willing and able to comply with the specified pre-operative and post-operative clinical and radiographic evaluations

The exclusion criteria are as follows:

- The patient has a history of total, unicompartmental reconstruction or fusion of the affected joint
- Patient has had a previous osteotomy around the knee
- The patient is morbidly obese (BMI > 41)
- The patient has a deformity which will require the use of stems, wedges or augments in conjunction with the Triathlon Total Knee System
- The patient has a varus/valgus deformity ≥ 15°
- The patient has a fixed flexion deformity ≥ 15°
- The patient has a neuromuscular or neurosensory deficiency, which would limit the ability to assess the performance of the device
- The patient has a systemic or metabolic disorder leading to progressive bone deterioration
- The patient is immunologically suppressed or receiving steroids in excess of normal physiological requirements
- Patient has a cognitive impairment, an intellectual disability or a mental illness
- The patient is unable to speak English
- The patient is pregnant
- The patient has metal hardware present in the region of the hip, knee or ankle (as this is known to create geometrical distortion in the region of the implant)

All patients will be screened by the orthopaedic consultant surgeon and research coordinator based on the criteria. Patients that meet these criteria and express an interest in participating will be provided an ethics approved patient information sheet following initial consultation with their treating doctor. This sheet provides more detail about the study, potential risks and requirements for follow-up. The research coordinator will assist in scheduling their pre-operative visits if the patient decides to participate in the study. Pre-operative visits include the collection of consent, CT scan, x-rays and completion of patient reported outcomes.

### Who will take informed consent? {26a}

Informed consent will be obtained by either the orthopaedic consultant surgeon or the research coordinator, both of whom are trained in the study requirements and will be appropriately onboarded. Consent will be collected at the pre-operative radiology visit which is scheduled up to 6 months before surgery, but normally occurs within 4 weeks of admission. Māori cultural support is also available as per the New Zealand ethics guidelines.

### Additional consent provisions for collection and use of participant data and biological specimens {26b}

Biological specimens are not collected as part of the study protocol and collection of participant data is incorporated into the consent process listed in the “Who will take informed consent? {26a}” section.

### Interventions

#### Explanation for the choice of comparators {6b}

All participants will undergo robotic arm-assisted TKA to control for surgical induced variability with the technology. MA is defined as the standard surgical technique in TKA, but its standardised approach is hypothesised to be a contributing factor to poorer functional outcome in some patients. FA is an individualised technique in TKA that aims to improve functional outcomes, but this is yet to be proven in an RCT. The high precision of robotic arm-assisted TKA will assist the surgeon in achieving both of the allocated surgical techniques.

#### Intervention description {11a}

All participants will undergo a pre-operative supine CT scan of the lower limb which will be loaded onto the robotic arm-assisted system to assist with planning, soft tissue assessment, ligament balancing and bone resections. In particular, the native bone anatomy will guide the starting implant positions for both MA and FA. Femoral resection landmarks are referenced from the most prominent point of the distal femoral condyles and the most posterior point of the posterior femoral condyles, avoiding osteophytes. Similarly, tibial resection points are placed at the midpoint of each plateau, two-thirds posteriorly in the anteroposterior plane.

All surgeries will be performed using a midline skin incision and a medial parapatellar approach with the femoral and tibial arrays placed extra-articular using bicortical pins. The pre-operative CT scan will be matched to the computer model following a verification process which identifies bony anatomy intra-operatively. The software will identify the hip centre and ankle position to calculate limb alignment. The haptic window defined in the robotic arm-assisted system allows for preservation of a tibial bone island ensuring the posterior cruciate ligament is maintained. All patients will receive a fully cemented Triathlon cruciate retaining implant (Triathlon, Stryker, Kalamazoo, MI, USA) with patella resurfacing. The patella and tibial bearing surfaces will use highly crosslinked polyethylene (X3™, Stryker, Kalamazoo, MI, USA). All procedures will be planned for a 9 mm polyethylene insert allowing for 1 mm adjustments to maximise range of motion and avoid hyperextension or ligament laxity. Femoral and tibial sizing are optimised using the 3D information provided by the CT scan.

For participants randomised to MA, the implant positions will be planned perpendicular to the femoral and tibial mechanical axis and aim to restore a neutral limb alignment (± 1°). Femoral component rotation is set to the trans-epicondylar axis whilst the tibial component is aligned to Akagi’s line, which connects the medial border of the patellar tendon to the middle of the posterior cruciate ligament [30]. In the sagittal plane, the femoral component is flexed between 0 and 5° to optimise implant size and prevent notching. The posterior slope is set to 0–3°, and a combined flexion limit (tibial + femoral component flexion) of 10° will be applied. Prior to any bone cuts, a manual varus and valgus stress is applied to the joint at 10° and 90° of flexion to provide a virtual gap assessment of ligament tension in the medial and lateral compartment. If balance cannot be achieved, then soft tissue releases will be performed by the surgeon.

For participants randomised to FA, the implant pre-operative plans will position the implants with equal medial and lateral resections of 6.5 mm from the subchondral bone of the femoral condyles to replicate the patient’s native anatomy. If present, bone wear is compensated for by adjusting the resection depth by 1–3 mm. Femoral rotation is therefore matched to the posterior condylar axis, and the tibial component is rotated to Akagi’s line. The proximal tibial resections will be set to 7 mm from subchondral bone in both the medial and lateral compartment. In the sagittal plane, the implants are positioned to match the patient’s native flexion and posterior tibial slope. Virtual gap assessment is then performed at 10° and 90° of flexion. The surgeon will then adjust implant position to achieve balance following FA principles [20], within set boundaries imposed on both coronal plane alignment and ligament laxities. Femoral component alignment is limited between 6° of valgus and 3° of varus, whilst tibial component alignment is limited to 6° of varus to 3° of valgus in the coronal plane, with an overall limb alignment target between 6° of varus and 3° of valgus. Gap balance is defined as an equal medial–lateral extension gap and equal gaps in the medial compartment from extension to flexion. A flexion gap differential in the lateral compartment up to 6 mm is permitted, as this represents the native laxity in the lateral flexion compartment and has been associated with improved patient outcomes [31]. Further, the implant used in this study is a single radius design and achieving isometric tension of the medial collateral ligament is thought to achieve a more natural pivot. An analytics tool is used to generate all possible balancing solutions based on surgeon defined alignment boundaries and balancing tolerances. A weighted scoring system assists the surgeon to select the optimal component alignment solution. The use of this algorithm also reduces surgical variability between all surgeons. If balance cannot be achieved within these boundaries, then soft tissue releases will be performed by the surgeon.

Intra-operative data will be collected from the robotic arm-assisted system, whilst in-patient rehabilitation data and discharge notes will be collected on the clinical research forms. Participants in both groups will follow the same in-patient post-operative rehabilitation programs and discharge criteria is based on the ability of the patient to mobilise with weight bearing and achieve a range of motion > 90°.

### Criteria for discontinuing or modifying allocated interventions {11b}

Both alignment techniques and the robotic arm-assisted system are already used for TKA at the site and the investigating surgeons will have overarching responsibility on the allocation of treatment for each participant. If a knee joint is unable to be satisfactorily balanced following the randomised intervention, then the surgeon may choose to proceed outside this protocol. The participant will then be excluded from the analysis and will follow the standardised care pathway. These patients will be identified as *intention to treat* in the final analysis.

### Strategies to improve adherence to interventions {11c}

As the intervention relates to surgical technique, all strategies are focused on patient management intra-operatively. Investigating surgeons will be informed of the randomised intervention 1 day prior to surgery and pre-operative planning will be conducted an hour before analgesia. In the FA group, the final implant positions will be based off soft tissue assessment during the procedure and verified post-operatively using data from the robotic arm-assisted system. In both groups final limb alignments will also be verified using post-operative long leg weight bearing x-rays.

### Provisions for post-trial care {30}

All participants will continue standard post-operative care with their surgeon at their conclusion of the trial. The sponsor, Stryker New Zealand Ltd, has met the Health and Disability Ethics Committee required for up-to-date insurance for injuries occurring as a result of participation in the trial. Compensation for other injuries will be covered under the New Zealand Accident Compensation Act (2001).

### Outcomes {12}

All participants will undergo assessment by the research team pre-operatively, and at 6 weeks, 6 months, 12 months and 24 months post-operatively. The research team will assist in the randomisation process and cannot be blinded to the allocation. The FJS, OKS, IKSS, KOOS, EQ-5D-5L, Net Promoter, BPI, PSQ and VAS pain are all validated clinical assessments of patients undergoing TKA [10, 32–38]. Physiotherapists will also record functional measures of recovery during in-patient stay and will be blinded to the allocated alignment. The functional measures were selected from recommended list as described by Dobson et al. [39] and include: 4 X 10 m walk test, passive and active range of motion, 30-s sit-to-stand test and a 3-step stair ascend and descend test. A breakdown of outcome measures is displayed in Table 1.

**Table 1:** Participant evaluation schedule

Evaluation	History/pre-op	Intra-op	In-patients	6 weeks	6 months	12 months	24 months
Demographics	X						
Medical history	X						
CT scan	X						
Surgical details		X					
Physiotherapy functional tests			X	X			
Recovery				X			
BPI	X		X	X			
PSQ	X						
Satisfaction					X	X	X
Net promoter					X	X	X
OKS	X			X	X	X	X
IKSS	X						X
FJS-12				X	X	X	X
KOOS	X						X
EQ-5D-5L	X			X	X	X	X
VAS pain	X			X	X	X	X
AP and ML X-ray	X						X
Long leg X-rays	X					X	

### Participant timeline {13}

Participants will be recruited from North Shore Hospital, Auckland and the Elective Surgery Centre, Auckland. Based on other randomised control trials performed at this site [40], this trial aims to recruit 12 patients per month. The recruitment process is estimated to take 22 months and began in November 2020. Results are anticipated in December 2025.

### Sample size {14}

This trial seeks to determine if robotic arm-assisted TKA following FA principles provides superior clinical outcomes to robotic arm-assisted TKA following MA principles. Ingelsrud et al. [41] reported that the minimal clinically important change in FJS for TKA patients was 14 points. As there is limited published literature on FA performed with robotic arm-assistance, the power calculations have assumed an effect size of 14, which represents a measure of superiority. A standard deviation of 26 is estimated using the Spearman correlation of 0.61 between the anchor score and change score from patients classified as ‘somewhat better’, which is also taken as a measure of superiority. Using a power of 80% (*β* = 0.2), significance level of 5% (*α* = 0.05) and accounting for 10% loss to follow-up yields a sample size of 120 participants per arm.

### Recruitment {15}

This trial includes five high volume arthroplasty surgeons from a large public teaching institution in Auckland. The orthopaedic consultant surgeons and research team will screen potential participants from the hospital waitlist. Participants that meet the eligibility criteria and express interest in participating will be provided with a patient information sheet. The research team will then telephone potential participants to confirm if they would like to enrol in the study. Patients were recruited face-to-face at a radiology appointment and will be recruited remotely during COVID-19 lockdowns.

### Assignment of interventions: allocation

#### Sequence generation {16a}

Participants will be recruited in a block size of 4:1, which aims to maintain balanced treatment arms over time. A master randomisation sequence will be generated prior to the start of the trial using an online random number generator (www.sealedenvelope.com). The master sequence will be maintained by the sponsor and patients will be allocated a treatment in sequential order following consent. The study team and surgeon will be notified of allocation prior to surgery.

#### Concealment mechanism {16b}

The sponsor will maintain the master randomisation sequence and will email the allocation treatment to the research team as each patient provides consent to participate in the trial.

#### Implementation {16c}

The research team will email the sponsor with the unique de-identified patient study number once a patient has been enrolled. The research team will inform the Investigating surgeons of the randomised intervention one day prior to surgery.

### Assignment of interventions: blinding

#### Who will be blinded {17a}

The participants and physiotherapists will be blinded to the treatment allocation. All participants will be assigned a unique study number following a consecutive order of consent. The participant identification list will be archived at the site on a secure network in a password-protected file. The investigators and research team are unable to be blinded to the allocation due to their role in the allocation of treatment and execution of surgery.

#### Procedure for unblinding if needed {17b}

Participants will be unblinded to the intervention at the end of the trial, unless there is a medical reason to do so prior to the end of the study.

### Data collection and management

#### Plans for assessment and collection of outcomes {18a}

Outcomes will be primarily captured and stored using a password-protected electronic platform (OBERD, Columbia, MO, USA). Participants will be sent automatic email reminders to complete their questionnaires. In the instance of non-compliance, the study coordinators will provide paper case report forms. The physiotherapists are also provided with an instruction manual and equipment to standardise the functional measures, which are collected on paper case report forms.

#### Plans to promote participant retention and complete follow-up {18b}

Electronic data capture will provide participants with the flexibility to complete the case report forms at their own convenience. Travel compensation for non-standard of care visits such as radiology appointments will also be provided to ensure retention is maintained. Participants who are classified as *intent to treat* but do not meet the surgical criteria will continue to be followed-up for outcome and safety purposes.

#### Data management {19}

The International Council for Harmonisation guidelines for Good Clinical Practice (ICH GCP) will be followed throughout the study. The sponsor will conduct routine monitoring visits for data verification against source material, as defined by participant entered data or medical records. Data collected by the sponsor will be stored electronically in a password-protected folder with restricted user access. Periodic surgeon investigator meetings will also be held to review participants where treatment has deviated from the protocol. The chief investigator will be responsible for the training and sign-off of all staff working on the study.

#### Confidentiality {27}

All research staff and investigators will be employed by the District Health Board and will comply with its confidentiality practices. All participants will be allocated a unique non-identifiable study number and any information disseminated in journals or conferences will ensure patient anonymity is maintained.

#### Plans for collection, laboratory evaluation and storage of biological specimens for genetic or molecular analysis in this trial/future use {33}

There is no planned collection of biological specimens for this study.

### Statistical methods

#### Statistical methods for primary and secondary outcomes {20a}

The primary and secondary outcome data gathered from patients in each study group will be pooled and summarised. The mean, standard deviation and 95% confidence intervals will be calculated for each measure in each group. All outcome measures will be assessed on their distribution and homogeneity to confirm the appropriate statistical model. A mixed-effects linear model will be used to compare longitudinal outcomes between the FA TKA (intervention) to the MA TKA (control), with pre-operative measures as a covariate. Adjustments will be made for multiple testing over time. Pairwise comparisons will be examined using a paired *t*-test if normally distributed or non-parametric test for skewed distribution. Categorical data will be evaluated using frequency and percent distributions, with significance testing performed using Fisher exact test or chi-squared test. Statistical significance is defined as a *p*-value < 0.05. Clinical significance will be assessed against the MCID and the PASS as defined in Orr et al. [42].

#### Interim analyses {21b}

Interim analysis will be conducted at milestones of 6-month and 1-year follow-up for dissemination of results. Periodic analysis will also be conducted for patient safety.

#### Methods for additional analyses (e.g. subgroup analyses) {20b}

Sub-group analysis based on patient phenotype will be dependent on sufficient post hoc sample size calculations.

#### Methods in analysis to handle protocol non-adherence and any statistical methods to handle missing data {20c}

A *per-protocol* and *intention to treat* analysis will be performed. In the event of randomisation errors, the participant will be converted to the study arm that represents the received treatment. The statistical model will be corrected in the event of missing data and to avoid type I error when performing multiple comparisons.

#### Plans to give access to the full protocol, participant level-data and statistical code {31c}

The full protocol is described here and will be summarised in future publications. Only the sponsor and site will have access to the participant-level dataset. The datasets analysed during the current study and statistical code are available from the corresponding author on reasonable request for the purpose of meta-analysis.

### Oversight and monitoring

#### Composition of the coordinating centre and trial steering committee {5d}

The sponsor will routinely monitor the progress of the trial and be responsible for the maintenance of ethics correspondence, governance and compliance documentation and data management. The principal investigator will be responsible for execution of GCP, delegation of authority to research staff and will review adverse events and protocol deviations. A trial steering committee will consist of the principal investigator, co-investigators, research coordinators/assistants and head physiotherapist. Routine meetings will be established to review any major adverse events or protocol deviations. A project sponsor team will also meet quarterly to review progress.

#### Composition of the data monitoring committee, its role and reporting structure {21a}

The principal investigator will review all adverse event forms for safety assessment. Any device or treatment related adverse events will be reviewed regularly by the Investigator team and a midpoint assessment of patient report outcomes will be conducted.

#### Adverse event reporting and harms {22}

An adverse event (AE) is defined as any undesirable clinical occurrence in a participant, whether it is considered to be device related or not, that includes a clinical sign, symptom or condition and/or an observation of an unintended technical performance or performance outcome of the device. A serious adverse event (SAE) is an adverse event that results in hospitalisation or prolongation of existing hospitalisation, persistent or significant disability or incapacity, life-threatening or death. All SAEs will be reported directly to the sponsor after review by the principal investigator for severity, seriousness and relationship to the surgical technique or device. Any event that is potentially associated with the surgical technique or device shall be reported to the local regulatory authority (MedSafe NZ) and the Health and Disability Ethics Committee by the sponsor. All series adverse events will be periodically examined using an alert in the participants electronic medical records and will be reviewed periodically in-line with the evaluation schedule.

#### Frequency and plans for auditing trial conduct {23}

The sponsor will conduct routine monitoring visits through the duration of the study. Yearly progress reports will be provided to the Northern B Health and Disability Ethics Committee.

#### Plans for communicating important protocol amendments to relevant parties (e.g. trial participants, ethical committees) {25}

Annual progress reports will be submitted to the Northern B Health and Disability Ethics Committee. Any substantial changes to the protocol or consent will be communicated to participants at their next follow-up visit. All investigators will be informed of future amendments and are required to sign-off on the current version of the protocol.

#### Dissemination plans {31a}

The results of this study will be published in peer-reviewed journals and presented at orthopaedic scientific conferences. Authorship will reflect contribution to the study and interpretation of the results. The principal investigator is responsible for dissemination of the results and the sponsor may only review and offer recommendations to the final wording. Participants will be informed about the results of the trial if they have ticked this option in the consent form.

## Discussion

CT-based robotic arm-assisted surgery is a useful tool to examine surgical alignment philosophies as it provides a wealth of information on patient morphology, as well as high precision to achieve the surgeon defined implant position. This study will utilise this technology to examine MA TKA and FA TKA. To our knowledge, this is the first randomised control trial to compare these two surgical techniques following the expanded alignment boundary limits in the coronal, sagittal and axial planes as described above. It is also unique by the fact that an advanced analytics tool is utilised to assist the surgeon in achieving balance. The multi-surgeon approach, consistency in surgical technique and post-operative care protocols, use of high precision surgical tools and the blinded randomisation will provide low bias in the results. We believe this study will yield high quality evidence to determine the optimal alignment technique to improve patient outcomes in TKA.

## Trial status

This is protocol version 3.0 dated 9 April 2021. Recruitment began in November 2020, and two amendments were submitted regarding the wording in the patient information, consent and patient evaluation schedule following the first 10 patients screened by the lead investigator. Recruitment is estimated to be completed by June 2022.

## Data Availability

Data will be stored securely in a password-protected file on a secure network and is available to all investigators at the site. De-identified line-item data will also be stored on a password-protected cloud server maintained by the sponsor. The final trial data for this protocol can be supplied on request for the purpose of meta-analysis.

## Abbreviations

AE: Adverse event
aMA: Adjusted mechanical alignment
BPI: Brief pain inventory
CT: Computer tomography
FA: Functional alignment
FJS: Forgotten joint score
GCP: Good clinical practice
IKSS: International Knee Society Score
KOOS: Knee Injury and Osteoarthritis Outcome Score
KA: Kinematic alignment
MA: Mechanical alignment
MCID: Minimum clinically important difference
OA: Osteoarthritis
OKS: Oxford Knee Score
PASS: Patient acceptable symptom state
PSQ: Pain Sensitivity Questionnaire
RCT: Randomised control trial
SAE: Serious adverse event
TKA: Total knee arthroplasty
VAS: Visual analogue scale
